# Acute vascular effects of vascular endothelial growth factor inhibition in the forearm arterial circulation

**DOI:** 10.1097/HJH.0000000000002230

**Published:** 2019-08-19

**Authors:** Alan C. Cameron, Paul Welsh, Karla B. Neves, David E. Newby, Rhian M. Touyz, Ninian N. Lang

**Affiliations:** a BHF Glasgow Cardiovascular Research Centre, Institute of Cardiovascular and Medical Science, University of Glasgow, Glasgow; b BHF Centre for Cardiovascular Sciences, University of Edinburgh, Edinburgh, UK

**Keywords:** bevacizumab, endogenous fibrinolysis, endothelin-1, endothelium-dependent vasodilatation, forearm arterial vasomotor function, hypertension, neoangiogenesis, vascular endothelial growth factor inhibitor

## Abstract

Although vascular endothelial growth factor inhibition (VEGFi) represents a major therapeutic advance in oncology, it is associated with hypertension and adverse vascular thrombotic events. Our objective was to determine whether VEGFi caused direct vascular dysfunction through increased endothelin-1 (ET-1) activity or impaired endothelial vasomotor or fibrinolytic function.

**Methods:**

Using forearm venous occlusion plethysmography, we measured forearm blood flow during intra-arterial infusions of bevacizumab (36–144 μg/dl forearm volume per minute) administered for 15–60 min in healthy volunteers (*n* = 6–8). On two separate occasions in 10 healthy volunteers, we further measured forearm blood flow and tissue plasminogen activator (t-PA) release during intra-arterial bradykinin infusion (100 and 1000 pmol/min) in the presence and absence of bevacizumab (144 μg/dl forearm volume per minute), and the presence and absence of endothelin A receptor antagonism with BQ-123 (10 nmol/min). Plasma t-PA and plasminogen activator inhibitor-1 (PAI-1) concentrations were measured at baseline and with each dose of bradykinin.

**Results:**

Baseline blood flow and plasma ET-1, t-PA and PAI-1 concentrations were unaffected by bevacizumab. Bradykinin caused dose-dependent vasodilatation (*P* < 0.0001) and t-PA release (*P* < 0.01) but had no effect on plasma PAI-1 concentrations. Neither bevacizumab nor BQ-123 affected bradykinin-induced vasodilatation and t-PA release.

**Conclusion:**

Acute exposure to bevacizumab does not directly cause endothelial vasomotor or fibrinolytic dysfunction in healthy young volunteers.

## INTRODUCTION

Vascular endothelial growth factor (VEGF) plays a key role in tumour growth and metastasis by stimulating neoangiogenesis, the formation of new blood vessels from pre-existing vasculature [1]. VEGF inhibition (VEGFi) is a major therapeutic advance for patients with previously untreatable cancers. Bevacizumab, the first VEGFi to be approved for clinical use, is a monoclonal antibody against VEGF. Small molecule VEGF tyrosine kinase inhibitors represent the other main class of VEGFi (e.g. sunitinib, pazopanib). However, these drugs are frequently associated with cardiovascular toxicities, of which systemic hypertension is the most common [1–4]. Almost every trial of VEGFi reports a treatment-associated increase in blood pressure (BP) and up to 80% of patients develop hypertension [1–3,5,6]. This can be difficult to treat and may limit the use of this therapy. VEGFi also increases the risk of arterial [7] and venous [8] thrombotic events over and above the pro-coagulant effects of malignancy [1,7].

Mechanisms underlying the unwanted vascular effects of VEGFi remain incompletely defined. Acute impairment of endothelium-dependent vasodilatation to acetylcholine has previously been demonstrated following brief exposure to a locally active dose of intra-arterial bevacizumab in the forearm arterial circulation of healthy volunteers [9]. Furthermore, plasma concentrations of endothelin 1 (ET-1), a potent vasoconstrictor, increase after treatment with VEGFi [10–14] and ET-1 receptor (ETR) antagonism prevents VEGFi-induced BP rise in animals [12,15,16]. Current anti-hypertensive therapies used for the treatment of patients with VEGFi-associated hypertension are generic and not pathophysiologically targeted.

The risk of thrombosis associated with VEGFi may relate to altered fibrinolysis. Tissue plasminogen activator (t-PA) is a serine protease that regulates fibrinolysis and is a marker of endothelial function. Its actions are counterbalanced by plasminogen activator inhibitor type 1 (PAI-1), a serpin that prevents excessive systemic fibrinolytic activity. The overall balance of fibrinolysis is thus important for the prevention of intravascular thrombosis and predicts the future risk of cardiovascular events [17].

We investigated the acute vasomotor and endogenous fibrinolytic effects of locally administered bevacizumab in the forearm arterial circulation of healthy volunteers. Specifically, we assessed dose-dependent effects of bevacizumab on resting vascular tone and bradykinin-induced endothelium-dependent vasodilator and endogenous fibrinolytic responses. With the aim of assessing ET-1 as a potential mediator of VEGFi-induced vascular actions, these assessments were made in the presence and absence of concurrent endothelin receptor antagonism.

## METHODS

The clinical study protocol (NCT03557190) was approved by the West of Scotland Research Ethics Committee 4 and carried out in accordance with the principles of the Declaration of Helsinki. Written informed consent was obtained from all participants prior to the study.

### Study participants

Healthy male volunteers were studied to avoid the potential for menstrual cycle-related changes in vascular function and to avoid potential latent teratogenicity in case of systemic spill-over of bevacizumab. Exclusion criteria included cardiovascular disease, hypertension, hyperlipidaemia, cerebrovascular disease, ischaemic heart disease, heart failure, atrial fibrillation/flutter, prior thrombotic/thromboembolic event, renal failure or hepatic failure, chronic medication, cigarette smoking, recreational drug use, history of anaemia, cancer, diabetes or macular degeneration, ongoing inflammatory, infective or autoimmune disease, live vaccination in the past 3 months or expected in the next 6 months, and BMI greater than 35 kg/m^2^.

### Forearm venous occlusion plethysmography

All studies were performed by a single investigator with the patient lying supine in a quiet, temperature-controlled (22–24 °C) room. Participants fasted for 4 h, abstained from alcohol for 24 h and did not consume any medications for 3 days before each study. Venous cannulae (17 gauge) were inserted into large subcutaneous veins of both antecubital fossae to facilitate venous sampling.

Brachial artery cannulation was performed using a 27-standard-wire-gauge steel needle. The total intra-arterial infusion rate was maintained at 1 ml/min throughout all studies. Forearm blood flow was measured in the infused and noninfused arms by venous occlusion plethysmography using mercury-in-silastic strain gauges (Hokanson EC4; Hokanson, Inc., Bellevue, Washington, USA), as described previously [18,19]. Supine heart rate and blood pressure were monitored at intervals throughout each study using a semiautomated, noninvasive oscillometric sphygmomanometer. The first three participants in each phase of the study returned for clinical review and repeat blood tests (full blood count, renal function/electrolytes, liver function tests, coagulation screen and glucose) to ensure safety and all other participants were contacted by telephone the day after each visit.

### Drugs

Pharmaceutical grade bradykinin (Bachem AG, Switzerland), BQ-123 (Bachem AG, Bubendorf, Switzerland) and bevacizumab (Roche Pharma AG, Grenzach-Wyhlen, Germany) were dissolved in physiological saline.

### Intra-arterial drug administration

#### Protocol 1: effects of vascular endothelial growth factor inhibition on resting forearm blood flow in healthy males

**FIGURE 1:**
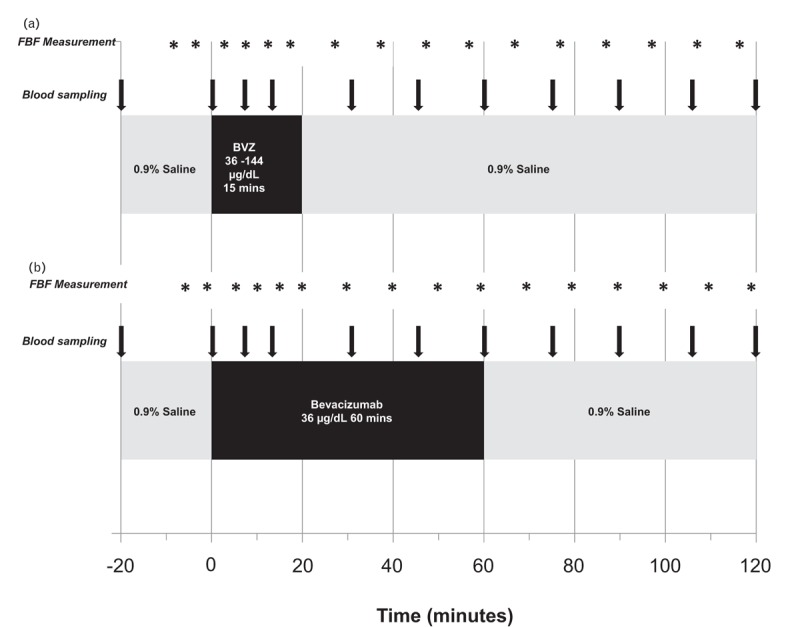
Overview of intra-arterial infusions and blood sampling. Protocol 1: effects of VEGFi on resting forearm blood flow; (a) bevacizumab 36, 72 or 144 μg/dl forearm volume per minute for 15 min, (b) Bevacizumab 36 μg/dl forearm volume per minute for 60 min. FBF, forearm blood flow; BVZ, bevacizumab. VEGFi, vascular endothelial growth factor inhibition.

After a 20-min equilibration phase of intra-arterial 0.9% sodium chloride infusion, intra-arterial bevacizumab was infused for 15 min at a constant dose of 36, 72 or 144 μg/dl forearm volume per min (*n* = 6, 6 and 8, respectively; Fig. 1a). Bevacizumab administered intra-arterially for 15 min at 144 μg/dl forearm volume per minute produces local forearm arterial concentrations similar to the plasma concentrations of bevacizumab found in patients after a clinically relevant intravenous dose of 5 mg/kg bevacizumab [9]. A further eight participants received a 60-min infusion of bevacizumab delivered at 36 μg/dl forearm volume per minute to explore the effects of longer exposure (Fig. 1b). Forearm blood flow was measured at 4-min intervals during the infusion of bevacizumab and at 10-min intervals for a total of 120 min thereafter.

#### Protocol 2: effects of vascular endothelial growth factor inhibition on stimulated blood flow, endogenous fibrinolysis and the effect of endothelin-receptor antagonism in healthy men

**FIGURE 2:**
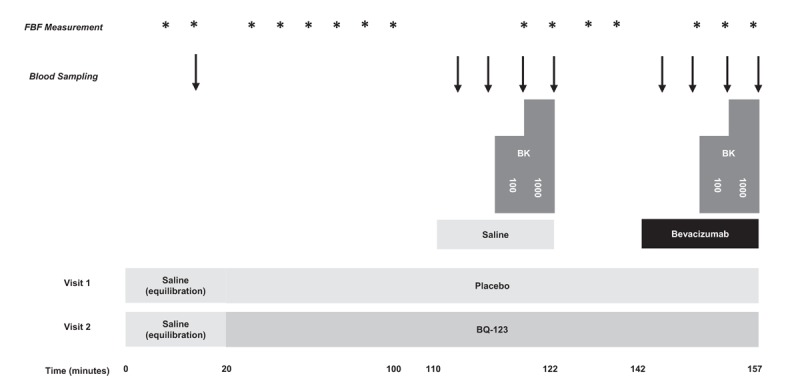
Overview of intra-arterial infusions and blood sampling. Protocol 2: the effect of VEGFi on stimulated forearm arterial vasodilatation and release of endogenous fibrinolytic factors. 100, 100 pmol/minute; 1000, 1000 pmol/minute; BK, bradykinin; BQ-123, BQ-123, 10 nmol/minute; FBF, forearm blood flow; bevacizumab, bevacizumab 144 μg/dl forearm volume per minute for 15 min; placebo, 0.9% saline. VEGFi, vascular endothelial growth factor inhibition.

A separate group of 10 healthy male volunteers attended for two visits, each separated by at least 14 days. Following a 20-min equilibration phase, bradykinin (100 and 1000 pmol/min), an endothelium-dependent vasodilator that stimulates endothelial t-PA release, was infused for 6 min at each dose, first in the absence and then in the presence of bevacizumab 144 μg/dl for 15 min. There was a 20-min washout between infusions. Double-blind randomization was performed at visit 1 to co-infusion of BQ-123 10 nmol/min (ET_A_R antagonist) or placebo (0.9% physiological saline), and the alternative agent was infused at visit 2. BQ-123 and placebo were infused for 90 min before bradykinin assessments to allow steady-state ET_A_R antagonism from BQ-123 [20] (Fig. 2).

### Blood sampling

Venous blood was collected at baseline for full blood count, renal function and electrolytes, liver function, lipid profile and glucose concentration. Blood samples were drawn from both arms at intervals during and after bevacizumab infusion, and with each dose of bradykinin, into serum tubes (BD Vacutainer, UK) for ET-1 assays, acidified buffered citrate (Stabilyte) tubes for t-PA assays and citrate tubes (BD Vacutainer) for PAI-1 assays. Samples were kept on ice before centrifugation at 2000 rpm for 10 min. Platelet-free plasma was decanted and stored at −80 °C before assay.

#### Measurement of plasma endothelin-1, tissue plasminogen activator and plasminogen activator inhibitor-1

ELISAs were performed to determine concentrations of endothelin-1 (human Endothelin-1 QuantiGlo ELISA Kit, QET00B; R&D Systems, Abingdon, UK), tissue plasminogen activator (Asserachrom t-PA, 00948; Stago, Reading, UK), plasminogen activator inhibitor-1 (PAI-1) antigen (Asserachrom PAI-1, 00949; Stago), and PAI-1 activity (Active Human PAI-1 functional assay kit, PI90; Oxford Biomedical Research, 2B Scientific, UK).

### Data analysis and statistics

Forearm plethysmographic data were analyzed as described previously [21]. The primary outcome was absolute forearm blood flow. Assessment of potential vasoconstrictor response was also analyzed as percentage change in forearm blood flow from baseline [22]. The estimated net release of plasma ET-1, t-PA and PAI-1 was defined as the product of the infused forearm plasma flow, based on haematocrit (Hct) and infused forearm blood flow, and the concentration difference between the infused and noninfused arms [21]. Variables are reported as mean ± SEM and were analysed using repeated measures ANOVA with post hoc correction for multiple comparison. Statistical analysis was performed using GraphPad Prism (GraphPad Software, San Diego, California, USA; Version 7) with statistical significance at 5%.

## RESULTS

### Participant characteristics

Thirty-eight healthy men aged between 18 and 52 years participated (mean age 25 years). Baseline demographics are presented in Table 1.

### Protocol 1: the effect of vascular endothelial growth factor inhibition on basal forearm arterial tone and the release of endothelin-1

#### Baseline forearm arterial blood flow

**FIGURE 3:**
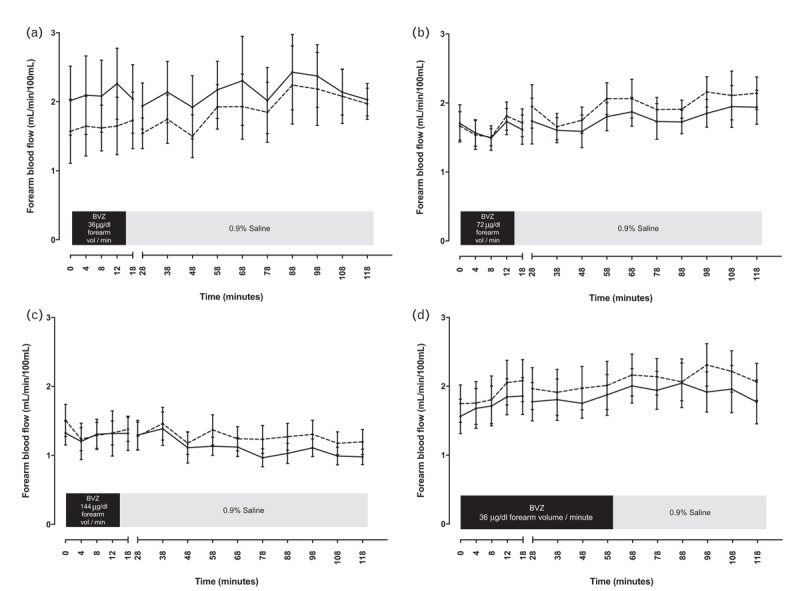
Absolute forearm blood flow in the infused arm (solid line) and noninfused arm (dashed line) during and after administration of bevacizumab. (a) 36, (b) 72 and (c) 144 μg/dl forearm volume per minute for 15 min, followed by 0.9% saline for 105 min and, (d) 36 μg/dl forearm volume per minute for 60 min, followed by 0.9% saline for 60 min. BVZ, bevacizumab; vol, volume.

Resting absolute forearm blood flow remained unchanged in the infused and noninfused arms when bevacizumab was administered at concentrations of 36, 72 and 144 μg/dl forearm volume per minute for 15 min, and 36 μg/dl forearm volume per minute for 60 min (*P* = NS for all; Fig. 3). When analysed as percentage change in forearm blood flow from baseline and as a ratio of infused : noninfused forearm blood flow, blood flow was again unchanged by bevacizumab at all doses and infusion durations (*P* = NS for all; data not shown).

#### The effect of vascular endothelial growth factor inhibition on endothelin-1 release

**FIGURE 4:**
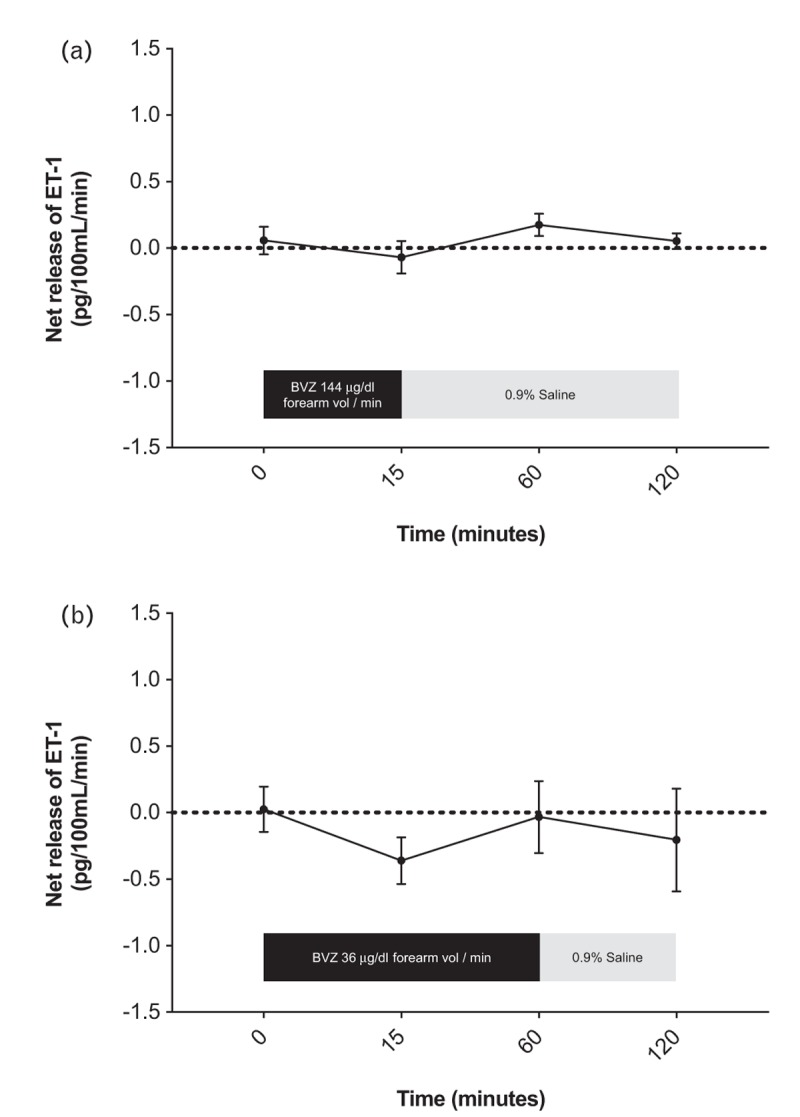
Net forearm endothelin-1 release during and after administration of bevacizumab. (a) 144 μg/dl forearm volume per minute for 15 min, followed by 0.9% saline for 105 min (*n* = 8) and (b) 36 μg/dl forearm volume per minute for 60 min, followed by 0.9% saline for 60 min (*n* = 8). ET-1, endothelin-1; BVZ, bevacizumab; vol, volume.

Baseline net ET-1 release was 0.06 ± 0.10 pg/100 ml/min and was unchanged during and after bevacizumab infusion at 144 μg/dl forearm volume per minute for 15 min (*P* = 0.24) or 36 μg/dl forearm volume per minute for 60 min (*P* = 0.42). There was no change in net ET-1 concentration at any time (*P* = NS for all, Fig. 4).

### Protocol 2: the effect of vascular endothelial growth factor inhibition on bradykinin-induced vasodilatation and release of fibrinolytic factors in the presence and absence of endothelin-receptor antagonism

#### The effect of vascular endothelial growth factor inhibition on bradykinin-induced vasodilatation

**FIGURE 5:**
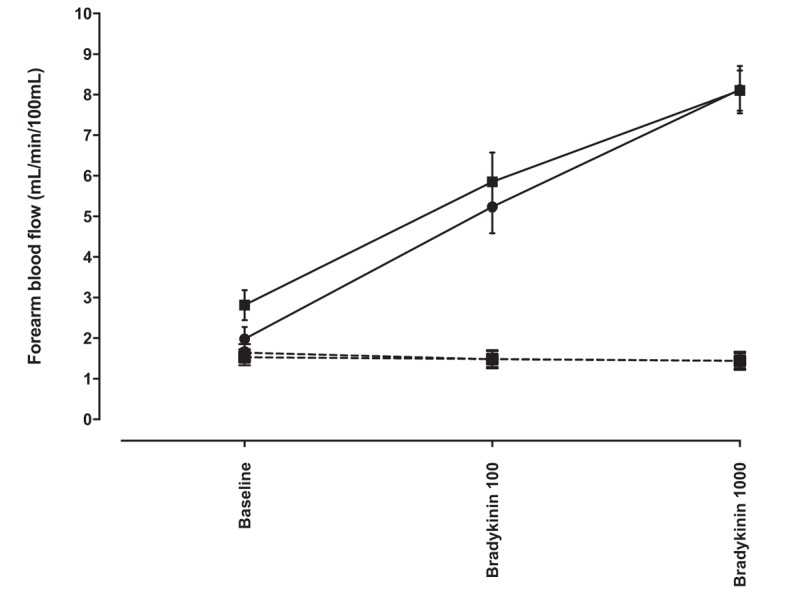
Absolute forearm blood flow in response to bradykinin 100 and 1000 pmol/minute in the presence and absence of bevacizumab 144 μg/dl forearm volume per minute for 15 min. Squares represent assessment in the presence of bevacizumab and circles represent assessment in the absence of bevacizumab (*n* = 10). Solid lines, infused arm; dashed lines, noninfused arm.

Intra-arterial bradykinin infusion evoked a dose-dependent vasodilatation (*P* < 0.0001; Fig. 5) that was unaffected by the presence of bevacizumab 144 μg/dl forearm volume per minute for 15 min (*P* = 0.45; Fig. 5).

#### The effect of vascular endothelial growth factor inhibition on bradykinin-induced tissue plasminogen activator and plasminogen activator inhibitor-1 release

Baseline net t-PA antigen release was −2.12 ± 3.65 ng/100 ml/min. This was unaffected by the presence of bevacizumab (*P* = 0.63). Bradykinin evoked dose-dependent release of net t-PA antigen (net t-PA antigen: 45.56 ± 11.06 ng/100 ml/min in the presence of bradykinin 100 pmol/min; 269.46 ± 58.47 ng/100 ml/min in the presence of bradykinin 1000 pmol/min; *P* < 0.01 for dose–response). This response was unaffected by the presence of bevacizumab (*P* = 0.49 for dose–response bradykinin-induced net t-PA Ag release in the presence versus the absence of bevacizumab). Net release of PAI-1 antigen and activity at rest were 0.22 ± 2.07 ng/100 ml/min and −2.41 ± 3.13 IU/100 ml/min, respectively. Bradykinin was not associated with a change in net PAI-1 antigen (*P* = 0.77) or activity (*P* = 0.14) and was unaffected by the presence of bevacizumab (*P* = NS for all).

#### The effect of endothelin-receptor antagonism on bradykinin-induced vasodilatation and fibrinolytic factor release in the presence of vascular endothelial growth factor inhibition

**FIGURE 6:**
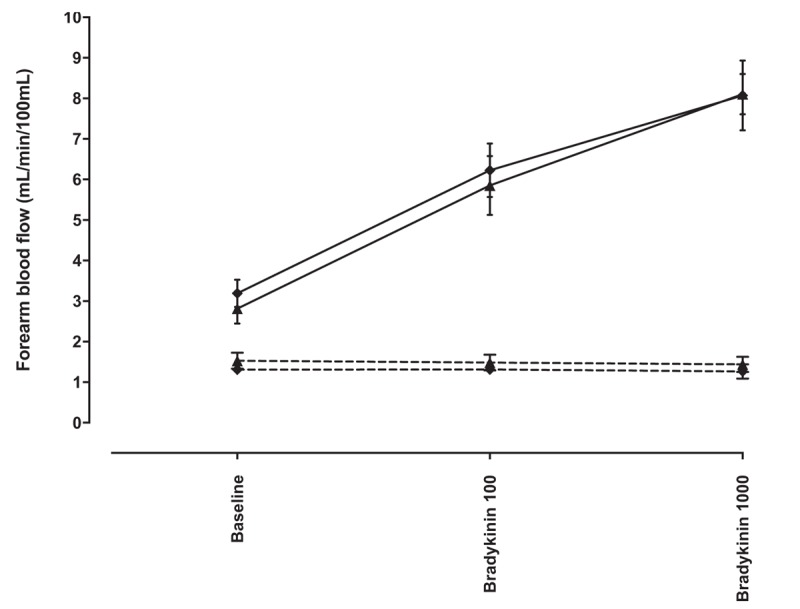
Absolute forearm blood flow in response to bradykinin 100 and 1000 pmol/min in the presence of intra-arterial bevacizumab 144 μg/dl forearm volume per minute for 15 min, with BQ-123 10 nmol per minute or placebo. Diamonds represent BQ-123 and triangles represent placebo. Solid lines, infused arm; dashed lines, noninfused arm.

Intra-arterial infusion of BQ-123 at 10 nmol/min increased forearm blood flow in the infused arm from 1.79 ± 0.19 to 2.32 ± 0.31 ml/100 ml/min at 80 min (*P* < 0.001). BQ-123 did not alter the vasodilator dose–response to bradykinin in the presence or absence of bevacizumab co-infusion (*P* = 0.73 and *P* = 0.52, respectively; Fig. 6). Dose-dependent bradykinin-induced t-PA antigen release was unaffected by BQ-123 in the presence of bevacizumab (*P* = 0.66). Net release of PAI-1 antigen and activity remained unchanged in response to bradykinin in the presence and absence of BQ-123 and irrespective of the presence or absence of bevacizumab (*P* = NS for all).

## DISCUSSION

We have comprehensively characterized the acute and direct effects of bevacizumab on endothelial vasomotor tone and endogenous fibrinolytic function using invasive sensitive gold standard measures of endothelial and vascular function. We have demonstrated that bevacizumab does not alter resting endothelial function or vascular tone and has no effect on plasma ET-1 concentrations. Moreover, VEGFi does not influence endogenous vasomotor or fibrinolytic responses to bradykinin. These data indicate that the VEGFi bevacizumab has no acute effects on vascular function, endothelial release of fibrinolytic factors and ET-1 production in healthy men.

Prior work examined the effect of a single acute dose of bevacizumab in the forearm arterial circulation and revealed no change in resting forearm arterial tone during intermittent assessment for 45 min [9]. In our study, bevacizumab also had no effect on resting arterial tone or net release of ET-1 for up to 2 h following its administration. We examined the effects of ascending doses of bevacizumab and the maximum dose given (144 μg/dl forearm volume per minute) produced a local forearm arterial concentration that reflects systemic concentrations achieved in patients treated with 5 mg/kg bevacizumab intravenously [9]. The vasoconstrictor effect of ET-1 is relatively slow in onset, taking at least 60 min for its full effects to manifest and plateau [23]. Therefore, by continuing observations for 2 h, we were able to make a more definitive conclusion that bevacizumab does not affect resting forearm vascular tone. Furthermore, by administering bevacizumab over a more prolonged period (1 h), we were unable to show that the increased time of vascular exposure to bevacizumab translates into a vasoconstrictor response in comparison to the shorter 15 min period of administration.

Forearm plethysmography is a robust method to measure vascular function in humans [18,24]. The current study was motivated by previous reports that endothelium-dependent vasodilatation in response to intra-arterial acetylcholine (ACh) is reduced by bevacizumab in a similar protocol to that used in our study [9]. In contrast to previous data, we did not observe impaired endothelium-dependent vasodilator response to bradykinin in the presence of bevacizumab. Important factors in this apparent discrepancy warrant discussion. First, a proportion of apparently healthy people are said to be acetylcholine ‘non-responders’ and were excluded from the final analysis of the previous study [9] and may have led to a type 2 error. However, bradykinin is much less susceptible to such inter-individual variation. Second, as the mechanisms of acetylcholine and bradykinin-induced vasodilatation may differ, VEGFi may differentially influence these mechanisms. Third, it is conventional to report and compare forearm arterial vasodilator effects as absolute blood flow rather than as a percentage change from baseline, which is associated with greater variability and can be affected disproportionately by small changes in baseline flow [25]. We present our data in terms of absolute blood flow responses. When analysed as percentage change from baseline or the ratio of absolute blood flow in infused : noninfused forearm, our results remain consistent. We, therefore, propose that our findings are valid.

It is of note that acetylcholine-evoked vasodilatation is mediated predominantly via nitric oxide (NO) [26]. In contrast, bradykinin invokes a greater relative contribution from endothelium-derived hyperpolarizing factor (EDHF) [27]. In addition to methodological differences, it is possible that the apparently different findings between our study and those of Thijs *et al* reflect this. Indeed, decreased NO bioavailability in the setting of VEGFi has been demonstrated in both preclinical models and in patients, and therefore, this may be more apparent when endothelial responses are assessed with acetylcholine than with bradykinin. However, our findings are also consistent with the observation that acetylcholine-mediated vasodilatation is unaffected in patients treated with sunitinib (a tyrosine kinase inhibitor of VEGF) for 1 week [28].

Unlike acetylcholine, bradykinin evokes the release of the endogenous fibrinolytic factor, t-PA. The assessment of this endothelial pathway is relevant not only as a sensitive and prognostic marker of vascular health [17] but is also relevant to assessing the putative endothelial mechanism underlying VEGFi-associated thrombosis. We demonstrate that although bradykinin is a potent agonist of t-PA release, this is not acutely affected by bevacizumab. Whether or not stimulated t-PA release is impaired in the setting of chronic exposure to VEGFi remains to be assessed, and it is also possible that older patients with co-morbidity and pre-existing endothelial dysfunction are more susceptible to effects of VEGFi. Given that both venous [8] and arterial [7] thromboembolism are increased in patients treated with bevacizumab, it is unlikely that this can be explained as a direct effect of arterial hypertension alone. In the context of the inflammatory and prothrombotic milieu of malignancy, relatively minor perturbations in the endogenous fibrinolytic pathway could contribute to VEGFi-associated thrombosis. Neither bradykinin nor bevacizumab affected the net release of PAI-1, the major endogenous inhibitor of t-PA activity.

In the absence of an effect of bevacizumab on endothelium-dependent vasodilatation, the conclusions that can be drawn from the lack of effect from endothelin receptor antagonism are constrained. It is clear, however, that BQ-123 does not potentiate bradykinin-evoked vasodilatation in the presence of bevacizumab in healthy people. However, BQ-123 has previously been demonstrated to increase acetylcholine-stimulated forearm arterial blood flow in patients with atherosclerosis but not in healthy people [29]. Therefore, the interplay between baseline cardiovascular risk and associated up-regulation of the ET-1 signalling pathway may mean that the situation is different in patients treated with VEGFi. Furthermore, up-regulation of ET-1 activity and bioavailability is a consistent feature of preclinical models of VEGFi and in cancer patients treated with these drugs [10–13]. BQ-123 is a selective ET-A receptor antagonist and we did not examine the effect of combined ET-A and ET-B receptor antagonism. Although the vasomotor effect of isolated ET-B activation is vasodilatation, the net effect of combined ET-A and ET-B activation remains to be potent vasoconstriction evoked via the strongly dominant effect of ET-A activation [30]. We believe it to be unlikely that additional ET-B antagonism would alter our findings.

Although we have not demonstrated an acute VEGFi-induced alteration in vasomotor activity, it remains plausible that ET-1 is up-regulated in patients following longer exposure to VEGFi, potentially in association with reduced NO bioavailability [9,31,32]. The chronic effects of VEGFi upon blood pressure may also reflect contributions from multiple pathogenic mechanisms including renal dysfunction, vascular rarefaction, oxidative stress and salt sensitivity [1,31,32].

The role of NO in vasomotor and fibrinolytic changes induced by VEGFi could be further explored by conducting assessments in the presence of the NO synthase inhibitor, N_ω_-Methyl-l-arginine acetate (l-NMMA). EDHF may be up-regulated in the setting of impaired NO bioavailability and the assessment of VEGFi upon EDHF-mediated vasomotor effects would be relevant. Further credence to this approach is provided by the observation that EDHF is exclusively responsible for bradykinin-induced t-PA release in patients with essential hypertension [33]. These assessments of the effects of VEGFi upon EDHF-mediated activity could be performed in the human forearm using intra-arterial sulfaphenazole, an inhibitor of cytochrome P450 2C9 that acts as an antagonist of EDHF.

Our studies have some limitations that should be considered. These assessments were conducted in healthy individuals where VEGF levels are not elevated and endothelial function is presumed to be normal. Thus, inhibiting VEGF signalling may be ineffective in this group and may not reflect the situation in patients with cancer or previous cardiovascular disease (CVD). We have no evidence of whether VEGF signalling was indeed inhibited by bevacizumab in the forearm vessels studied. The maximum dose of bevacizumab we administered achieves local forearm arterial concentrations comparable with the systemic concentration achieved with 5 mg/kg intravenous (IV) bevacizumab. This is a clinically applicable dose that is associated with hypertension in 42% of patients [34]. Although some treatment regimens employ up to 15 mg/kg of intravenous bevacizumab [35,36], we were reluctant to administer such large doses to the forearm arterial circulation with concern about the potential for systemic spill-over. We did not assess directly the effect of VEGFi upon the activity of other endothelial vasomotor pathways, including NO and EDHF. However, we wished to minimize exposure of healthy volunteers to bevacizumab and our primary focus was the assessment of ET-1 signalling in the context of VEGFi.

In conclusion, brief exposure to bevacizumab does not increase resting vascular tone and has no effect on bradykinin-induced vasorelaxation. Moreover, bevacizumab did not influence endothelial activity as evidenced by no effect on production of endothelial-derived fibrinolytic factors or endothelin-1. Future vascular assessment should focus on effects of longer term exposure to VEGFi in cancer patients. This will allow the identification of putative vascular mechanisms of VEGFi-associated hypertension and thrombosis in cancer patients treated with VEGFi.

## ACKNOWLEDGEMENTS

We would like to thank all volunteers for participating in the study and all members of the research team, including research nurses Ammani Brown and Laura Kelly, and laboratory manager Jackie Thomson, for their support.

Sources of funding: This study was supported by the Chief Scientist Office (CSO) Scotland [TCS/16/31] and the British Heart Foundation (BHF) [RE/13/5/30177 to R.M.T., BHF Chair CH/12/429762].

### Conflicts of interest

There are no conflicts of interest.

**TABLE 1:** Demographics of study participants

	Protocol 1: resting				Protocol 2: BK-stimulated
Infusion (s)	BVZ 36 μg/dl, 15 min	BVZ 72 μg/dl, 15 min	BVZ 144 μg/dl, 15 min	BVZ 36 μg/dl, 60 min	BVZ 144 μg/dl 15 min with BK ± BQ-123
Participants (*n*)	6	6	8	8	10
Age (years)	22.6 ± 1.9	29.8 ± 5.9	25.9 ± 2.3	24.7 ± 1.5	23.5 ± 1.5
Height (m)	177.7 ± 4.2	183.4 ± 2.1	176.4 ± 2.6	180.6 ± 1.9	179.3 ± 1.4
Weight (kg)	74.6 ± 4.2	79.6 ± 5.3	69.2 ± 2.9	69.7 ± 2.4	75.6 ± 2.3
BMI (kg/m^2^)	23.6 ± 1.1	23.4 ± 1.2	22.3 ± 1.1	21.4 ± 0.8	23.5 ± 0.7
SBP (mmHg)	122 ± 5	127 ± 3	124 ± 4	118 ± 3	122 ± 3.4
DBP (mmHg)	73 ± 3	77 ± 4	76 ± 3	67 ± 3	70 ± 3
Heart rate (bpm)	67 ± 3	60 ± 2	65 ± 4	59 ± 3	61 ± 3
Haemoglobin (g/l)	150 ± 6	159 ± 7	155 ± 2	148 ± 3.6	150 ± 3
Glucose (mmol/l)	4.7 ± 0.1	4.6 ± 0.3	4.6 ± 0.1	4.7 ± 0.2	4.6 ± 0.2
Cholesterol (mmol/l)	3.4 ± 0.7	5.5 ± 0.5	4.6 ± 0.5	3.9 ± 0.1	4.6 ± 0.4
Triglycerides (mmol/l)	1.0 ± 0.1	1.4 ± 0.2	1.0 ± 0.1	0.7 ± 0.1	0.9 ± 0.1

BVZ, bevacizumab; BK, Bradykinin; bpm, beats per minute.
